# Red blood cell distribution width-to-albumin ratio is a risk factor for all-cause and cardiovascular mortality in patients with CKM stages 1 to 4: Evidence from the NHANES 2007 to 2016

**DOI:** 10.1097/MD.0000000000045682

**Published:** 2025-11-07

**Authors:** Bing Wang, Shanshan Zhou

**Affiliations:** a Department of Cardiovascular Diseases, The First Hospital of Jilin University, Changchun, Jilin Province, China.

**Keywords:** all-cause mortality, cardiovascular mortality, CKM, Fine–Gray model, red blood cell distribution width–albumin ratio

## Abstract

Cardiovascular–kidney–metabolic (CKM) syndrome is a systemic condition driven by inflammation. The red blood cell distribution width-to-albumin ratio (RAR) has emerged as a novel marker of systemic inflammation, but its prognostic value in CKM remains unclear. We analyzed 9135 participants from National Health and Nutrition Examination Survey 2007 to 2016 with mortality data obtained from the National Death Index. Weighted Cox proportional hazards models assessed the association between RAR and mortality. Generalized additive models, time-dependent receiver operating characteristic curves, and Fine–Gray competing risk models were used for further evaluation. Over a median follow-up of 91.95 months, 987 deaths occurred, including 241 cardiovascular deaths. Based on an optimal cutoff (RAR = 3.33), participants were stratified into higher and lower RAR groups. The weighted Cox proportional hazards model revealed that individuals with higher RAR had significantly increased risks of all-cause mortality (hazard ratio 2.30, 95% confidence interval [CI] 1.95–2.70) and cardiovascular mortality (hazard ratio 3.26, 95% CI 2.40–4.42). Generalized additive models demonstrated a positive association between RAR and mortality. The main findings remained consistent in the sensitivity analyses. The area under the curve values for predicting all-cause mortality at 1, 3, 5, and 10 years were 0.756, 0.717, 0.708, and 0.695, respectively; the corresponding area under the curves for cardiovascular mortality were 0.708, 0.729, 0.737, and 0.714, respectively. Furthermore, even after accounting for noncardiovascular deaths as competing risk factors, The Fine–Gray model revealed that RAR was an independent predictor of cardiovascular mortality (subdistribution hazard ratio 1.68, 95% CI 1.38–2.04). Elevated RAR independently increases the risk of all-cause and cardiovascular mortality in patients with CKM stages 1 to 4.

## 1. Introduction

Cardiovascular–kidney–metabolic syndrome (CKM) is a pathophysiologically interconnected condition that affected >25% of U.S. adults between 2015 and 2020 and was a leading cause of death in 2021.^[1]^ CKM syndrome is associated with an increased risk of all cardiovascular disease (CVD) phenotypes, including coronary artery disease, stroke, heart failure, peripheral arterial disease, atrial fibrillation, and sudden cardiac death.^[2–6]^ Moreover, owing to the intrinsic interconnections among its components, many patients with CKM syndrome present with multiple comorbidities, resulting in significantly elevated mortality rates.^[7]^ The American Heart Association has proposed classifying CKM syndrome into 5 stages (0–4), where stage 0 represents individuals without CKM risk factors,^[8]^ and mortality risk progressively increases with increasing CKM stage.^[9]^ A nationally representative study based on National Health and Nutrition Examination Survey (NHANES) data reported that between 2011 and 2020, approximately 89.4% of US adults were classified into CKM syndrome stages 1 to 4, with only 10.6% having no CKM risk factors (stage 0).^[1]^ Therefore, identifying the risk factors for all-cause and cardiovascular mortality in a large population of patients with CKM stages 1 to 4 is both urgent and critically important.

CKM syndrome is a clinical manifestation of the pathophysiological interplay among metabolic risk factors such as obesity, diabetes, chronic kidney disease (CKD), and cardiovascular disease.^[7]^ Numerous studies have demonstrated that excessive adipose tissue can trigger systemic inflammation,^[10–12]^ which in turn contributes to the development of cardiovascular events. Therefore, inflammation plays a pivotal role in the occurrence of cardiovascular and CKM-related events. Red cell distribution width (RDW) is a routinely measured parameter in complete blood count tests that reflects the heterogeneity of red blood cell size. Elevated RDWs may result from impaired erythropoiesis and abnormal red blood cell survival and are associated with inflammation, oxidative stress,^[13,14]^ and various diseases, such as diabetes,^[15]^ kidney disease,^[16]^ cardiovascular disease, heart failure, and mortality.^[17]^ Serum albumin, the most abundant circulating protein in the blood, possesses anti-inflammatory and antioxidant properties and serves as a key marker of inflammation.^[18]^ As both RDW and serum albumin levels are closely linked to inflammation and oxidative stress (albeit reflecting these pathologies from different perspectives) their integration holds significant value in predicting mortality risk.

A growing body of evidence has confirmed a significant association between the red cell distribution width-to-albumin ratio (RAR) and CKM-related risk factors such as kidney disease and cardiovascular disease.^[17,19–21]^ A recent analysis based on the NHANES demonstrated that RAR is significantly associated with mortality risk in the general population.^[22]^ However, the relationship between RAR and mortality risk among individuals with CKM stages 1 to 4 has not been fully elucidated. Given this background, investigating the association between RAR and mortality in individuals with CKM stages 1 to 4 holds substantial scientific and clinical significance.

## 2. Methods

### 2.1. Survey population

This study utilized data from the 2007 to 2016 cycles of the NHANES. The NHANES is a continuous cross-sectional survey conducted on the noninstitutionalized U.S. population that uses a multistage probability sampling design to collect health, nutritional, and demographic data from participants. The data used in this study were obtained from publicly available files and were approved by the National Center for Health Statistics Research Ethics Review Board. All participants provided written informed consent. This study adhered to the Strengthening the Reporting of Observational Studies in Epidemiology guidelines.

### 2.2. Study design and population

The participants included adults aged 20 years and older who participated in the NHANES from 2007 to 2016. During the screening process, individuals were excluded if they lacked data on RAR, CKM classification, survival status, or covariates or if they were classified as CKM stage 0. A total of 9135 eligible participants with CKM stages 1 to 4 were included in the final analysis (Fig. S1, Supplemental Digital Content, https://links.lww.com/MD/Q556).

### 2.3. CKM syndrome

CKM syndrome is a clinical manifestation of the pathophysiological interactions among metabolic risk factors, including obesity, diabetes, CKD, and cardiovascular conditions.^[7]^ Participants were classified into different CKM stages on the basis of their health status: stage 1: excess or dysfunctional adiposity or both, with dysfunctional adiposity defined as impaired glucose tolerance or prediabetes; stage 2: presence of additional metabolic risk factors (e.g., hypertriglyceridemia, hypertension, diabetes, metabolic syndrome) or moderate- to high-risk CKD; stage 3: subclinical CVD in the context of CKM syndrome or CVD risk equivalents (high predicted 10-year CVD risk or very high-risk CKD); stage 4: established clinical CVD (e.g., coronary artery disease) in CKM syndrome. The 10-year cardiovascular risk was estimated via the American Heart Association’s Predicting Risk of CVD EVENTs (PREVENT) equations.^[7]^ The base equations are provided in Table S1, Supplemental Digital Content, https://links.lww.com/MD/Q556. CKD classification was based on the Kidney Disease: Improving Global Outcomes guidelines, which use the estimated glomerular filtration rate (eGFR) and urine albumin-to-creatinine ratio.^[23]^ The eGFR was calculated via the 2021 Chronic Kidney Disease Epidemiology Collaboration creatinine equation, which does not include race or ethnicity.^[24]^ A more detailed description is available in Table S2, Supplemental Digital Content, https://links.lww.com/MD/Q556.

### 2.4. Definition of clinical outcomes

We acquired mortality data from the NHANES Public Use Linked Mortality File as of December 31, 2019, which employs a probabilistic matching technique to align with the National Death Index from the National Centre for Health Statistics. The cause of death was determined following the International Statistical Classification of Diseases, 10th Revision, and the specified outcome of death was reclassified. All-cause mortality refers to deaths attributable to any cause. Cardiovascular mortality refers to deaths caused by heart disease and cerebrovascular disease.^[25]^

### 2.5. Measurement of RAR

RAR was calculated as the ratio between RDW and serum albumin levels, which were measured via the bromocresol purple method and a Coulter® DxH 800 hematology analyzer (Beckman Coulter, Inc., Brea), respectively.

### 2.6. Covariates

The selection of covariates in this study was based on clinical expertise and informed by previous studies.^[22,26]^ The included covariates included age, gender, race, marital status, poverty income ratio (PIR), education level, physical activity, smoking status, alcohol intake, body mass index (BMI), waist circumference, eGFR, hypertension, diabetes, CVD, stroke, and hyperlipidemia. These covariates were extracted from the demographic, dietary, examination, laboratory, and questionnaire components of the NHANES database. Detailed definitions and coding of covariates are provided in Supplementary Materials, Supplemental Digital Content, https://links.lww.com/MD/Q556.

### 2.7. Statistical analysis

Statistical analyses were conducted via R software (version 4.4.1; R Foundation for Statistical Computing, Vienna, Austria) and EmpowerStats (version 4.2; X&Y Solutions, Inc., Boston ). Given that the NHANES adopts a stratified, multistage probability sampling design, the “survey” package was used to account for complex sampling weights. The “maxstat” package was employed to perform maximally selected rank statistics to determine the optimal cutoff value for RAR, classifying participants into lower- and higher-RAR groups.^[27]^ The means ± standard deviations of the continuous variables were calculated via survey-weighted linear regression (svyglm). Survey-weighted percentages for categorical variables were also calculated via survey-weighted linear regression (svyglm). Weighted Cox proportional hazards models were used to evaluate the independent associations between RAR and all-cause and cardiovascular mortality in patients with CKM stages 1 to 4. The results are presented as Model 1 (unadjusted), Model 2 (adjusted for age, gender, and race), and Model 3 (adjusted for age, gender, race, marital status, PIR, education level, physical activity, smoking status, alcohol intake, BMI, waist circumference, eGFR, hypertension, diabetes, CVD, stroke, and hyperlipidemia). Generalized additive models (GAMs) were conducted to visually explore the relationship between RAR and mortality. The sensitivity analyses included subgroup analyses; multiple imputation for missing data via the chained equations method, which generated 5 imputed datasets. Detailed procedures for multiple imputation are provided in Supplementary Methods, Supplemental Digital Content, https://links.lww.com/MD/Q556; analyses using unweighted data; analysis of the association between RAR and mortality after RAR is categorized into quartiles. The “timeROC” package was used to evaluate the time-dependent predictive accuracy of RAR for survival outcomes at multiple time points.^[27]^ Subdistribution hazard ratios (SHRs) and 95% confidence intervals (CIs) were calculated via the Fine–Gray competing risk model.

## 3. Results

### 3.1. Participant characteristics

**Figure 1.**
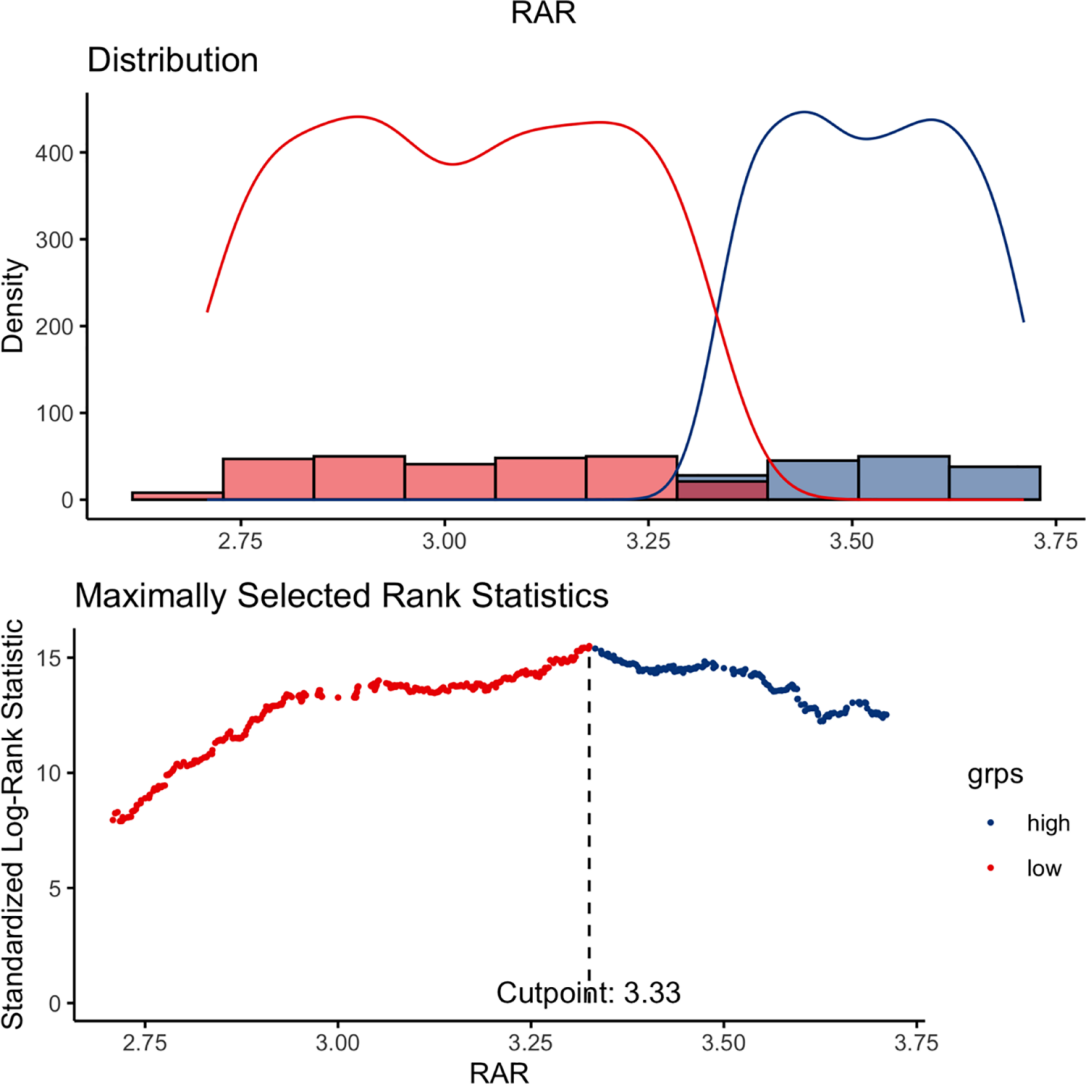
The cutoff point was calculated using the maximally selected rank statistics based on the “maxstat” package (R Foundation for Statistical Computing, Vienna, Austria).

As shown in Table 1, this study included 9135 participants with CKM stages 1 to 4 from the 2007 to 2016 NHANES cycles, representing the U.S. adult population after applying NHANES sampling weights. Using the maximally selected rank statistics, the optimal cutoff value of RAR associated with survival was determined to be 3.33. The participants were thus divided into a higher RAR group (RAR > 3.33, n = 2468) and a lower RAR group (RAR ≤ 3.33, n = 6667) (Fig. 1). Compared with those in the lower RAR group, the participants in the higher RAR group were older (54.70 years vs 49.61 years, *P* < .001). Significant differences (*P* < .05) were also observed between the 2 groups in terms of gender, race, education level, marital status, PIR, alcohol intake, physical activity, BMI, waist circumference, white blood cell count, neutrophil count, lymphocyte count, monocyte count, and platelet count, as well as comorbidities, including CVD, stroke, diabetes, and hypertension (Table 1).

**Table 1:** Characteristics of the NHANES 2007 to 2016 participants.

	Total (N = 9135)	Lower RAR (N = 6667)	Higher RAR (N = 2468)	*P* value
Age, mean ± SD	50.99 ± 17.23	49.61 ± 17.10	54.70 ± 17.06	**<.001**
Age (%)				**<.001**
≤60	72.94	75.62	63.45	
>60	27.06	24.38	36.55	
Gender (%)				**<.001**
Female	48.93	43.39	68.47	
Male	51.07	56.61	31.53	
Race (%)				**<.001**
Mexican American	8.30	8.39	8.02	
Other Hispanic	5.29	5.17	5.69	
Non-Hispanic White	70.12	72.89	60.36	
Non-Hispanic Black	9.91	6.96	20.31	
Other Race	6.38	6.60	5.62	
Education level (%)				**<.001**
High school or below	16.89	15.45	21.99	
Some college or AA degree	22.31	21.87	23.89	
College graduate or above	60.80	62.69	54.12	
Marital status (%)				**<.001**
Married	57.95	59.57	52.23	
Never married	15.64	15.74	15.26	
Living with partner	7.59	7.78	6.93	
Other	18.82	16.91	25.58	
PIR (%)				**<.001**
≤1.3	21.98	19.82	29.58	
1.3–3.5	36.14	35.16	39.60	
>3.5	41.89	45.02	30.82	
Smoking status (%)				.076
No	53.08	53.73	50.77	
Yes	46.92	46.27	49.23	
Alcohol intake (%)				**<.001**
No	22.57	20.03	31.55	
Yes	77.43	79.97	68.45	
BMI (kg/m^2^) %				**<.001**
≤25	21.95	23.63	16.01	
25–30	37.93	41.00	27.11	
>30	40.12	35.37	56.87	
Waist circumference (cm), mean ± SD	101.28 ± 15.48	99.22 ± 14.02	106.86 ± 17.72	**<.001**
eGFR (mL/min/1.73 m^2^)	95.70 ± 27.13	96.40 ± 24.89	93.83 ± 32.35	.720
Hypertension (%)				**<.001**
No	60.52	63.65	49.48	
Yes	39.48	36.35	50.52	
Hyperlipidemia (%)				.515
No	23.21	23.39	22.58	
Yes	76.79	76.61	77.42	
Physical activity (%)				**<.001**
No	27.60	24.29	39.28	
Yes	72.40	75.71	60.72	
Diabetes (%)				**<.001**
No	83.97	86.56	74.86	
Yes	16.03	13.44	25.14	
CVD (%)				**<.001**
No	92.38	93.79	87.39	
Yes	7.62	6.21	12.61	
Stroke (%)				**<.001**
No	96.79	97.59	93.97	
Yes	3.21	2.41	6.03	
WBC (×10^9^/L)	6.90 ± 2.64	6.72 ± 1.90	7.38 ± 3.97	**<.001**
Neutrophil (×10^9^/L)	4.04 ± 1.84	3.92 ± 1.53	4.35 ± 2.46	**<.001**
Lymphocyte (×10^9^/L)	2.06 ± 1.49	2.03 ± 0.64	2.17 ± 2.67	**<.001**
Monocyte (×10^9^/L)	0.54 ± 0.22	0.53 ± 0.18	0.57 ± 0.29	**<.001**
Platelet (×10^9^/L)	241.79 ± 66.19	237.63 ± 59.25	253.04 ± 81.00	**<.001**

*Note*: Mean ± standard deviation for continuous variables: *P* value was calculated by survey-weighted linear regression (svyglm). Survey-weighted percentages for categorical variables: survey-weighted linear regression (svyglm). Statistical significance was defined as *P* < .05. Bold values indicate comparisons that reached statistical significance.

BMI = body mass index, CVD = cardiovascular disease, eGFR = estimated glomerular filtration rate, NHANES = National Health and Nutrition Examination Survey, PIR = ratio of family income to poverty line, RAR = red blood cell distribution width-to-albumin ratio, SD = standard deviation, WBC = white blood cell.

### 3.2. Associations of the RAR with all-cause mortality

**Figure 2.**
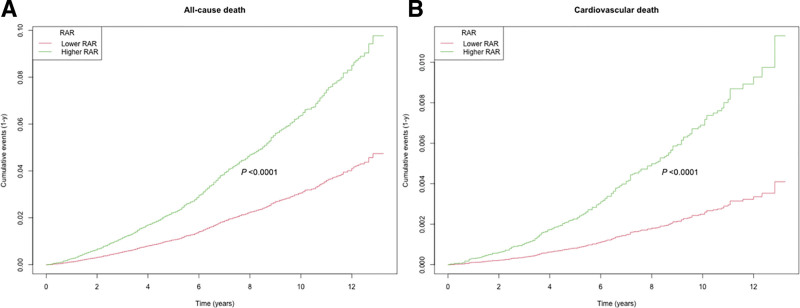
Kaplan–Meier curves of the cumulative incidence of death in patients with CKD stages 1 to 4, stratified by RAR values (>3.33 vs ≤3.33). The red line represents the RAR ≤ 3.33 group, while the green line represents the RAR > 3.33 group. (A) All-cause mortality. (B) Cardiovascular mortality. CKD = chronic kidney disease, RAR = red blood cell distribution width-to-albumin ratio.

**Figure 3.**
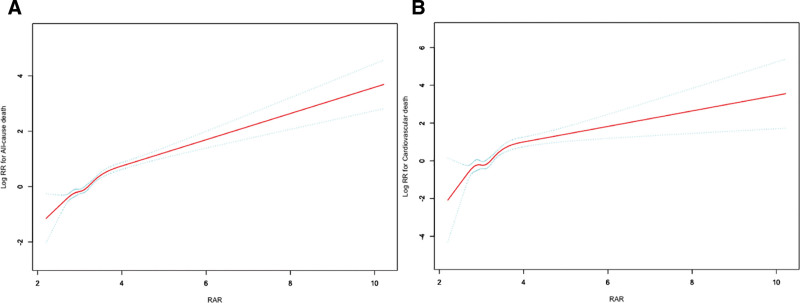
The association of RAR with all-cause (A) and cardiovascular mortality (B) among diabetes visualized by generalized additive models. Hazard ratios were adjusted for age, gender, race, marital status, education level, physical activity, smoking status, alcohol intake, BMI, waist circumference, eGFR, waist circumference, hypertension, diabetes, CVD, stroke, and hyperlipidemia. BMI = body mass index, CVD = cardiovascular disease, eGFR = estimated glomerular filtration rate, RAR = red blood cell distribution width-to-albumin ratio.

During a median follow-up period of 91.95 months, 987 out of 9135 participants with CKM stages 1 to 4 (10.80%) died, including 241 (2.64%) from cardiovascular causes. The Kaplan–Meier curves for all-cause and cardiovascular mortality revealed significantly different cumulative incidence rates between the higher- and lower-RAR groups (*P* < .0001), with higher mortality observed in the higher-RAR group (Fig. 2). In the unadjusted model (Model 1) of the weighted Cox proportional hazards analysis, we observed that higher RAR values were significantly associated with increased risks of both all-cause mortality (hazard ratio [HR] 2.22, 95% confidence interval [CI] 1.94–2.54, *P* < .0001) and cardiovascular mortality (HR 2.38, 95% CI 2.02–2.80, *P* < .0001) (Table 2). In the fully adjusted model (Model 3), each unit increase in RAR was associated with a 108% increase in all-cause mortality risk (HR 2.08, 95% CI 1.78–2.42, *P* < .0001) and a 141% increase in cardiovascular mortality risk (HR 2.41, 95% CI 1.92–3.03, *P* < .0001) (Table 2). The weighted Cox regression analysis further demonstrated that, compared with the lower RAR group, the higher RAR group had significantly elevated risks of all-cause and cardiovascular mortality according to the unadjusted model (all-cause: HR 3.34, 95% CI 2.84–3.93, *P* < .0001; cardiovascular: HR 4.54, 95% CI 3.42–6.04, *P* < .0001) to the fully adjusted model (Model 3) (all-cause: HR 2.30, 95% CI 1.95–2.70, *P* < .0001; cardiovascular: HR 3.26, 95% CI 2.40–4.42, *P* < .0001) (Table 2). Moreover, GAMs revealed a significant positive nonlinear association between RAR and both all-cause mortality and cardiovascular mortality among participants with CKM stages 1 to 4 (Fig. 3).

**Table 2:** Associations between RAR and mortality among participants with CKM stages 1 to 4.

	Model 1	Model 2	Model 3
	HR (95% CI) *P*	HR (95% CI) *P*	HR (95% CI) *P*
All-cause mortality			
RAR	2.22 (1.94–2.54) <.0001	2.42 (2.09–2.81) <.0001	2.08 (1.78–2.42) <.0001
RAR category			
Lower RAR	Reference	Reference	Reference
Higher RAR	3.34 (2.84–3.93) <.0001	2.92 (2.45–3.47) <.0001	2.30 (1.95–2.70) <.0001
Cardiovascular mortality			
RAR	2.38 (2.02–2.80) <.0001	2.72 (2.22–3.34) <.0001	2.41 (1.92–3.03) <.0001
RAR category			
Lower RAR	Reference	Reference	Reference
Higher RAR	4.54 (3.42–6.04) <.0001	4.09 (2.92–5.72) <.0001	3.26 (2.40–4.42) <.0001

Model 1: crude model without adjustment for any covariates. Model 2: adjustments for age, gender, and race; Model 3: adjustments for age, gender, race, marital status, education level, physical activity, smoking status, alcohol intake, PIR, BMI, waist circumference, hypertension, diabetes, CVD, stroke, and hyperlipidemia.

BMI = body mass index, CI = confidence interval, CKM = cardiovascular–kidney–metabolic, CVD = cardiovascular disease, HR = hazard ratio, RAR = red blood cell distribution width-to-albumin ratio.

### 3.3. Sensitivity analyses

We first conducted subgroup analyses stratified by age, sex, smoking status, BMI, hypertension status, and diabetes status to explore the associations between RAR and both all-cause mortality and cardiovascular mortality. The core findings remained consistent across all subgroups. Furthermore, no significant interactions were observed between RAR and the stratification variables (*P* for interaction > .05), indicating the robustness of our results (Table S3, Supplemental Digital Content, https://links.lww.com/MD/Q556). In addition, we performed multiple imputation, unweighted Cox regression analysis, and quartile-based analyses of RAR. All these sensitivity analyses yielded consistent and significant results, further confirming the robustness of the association between RAR and both all-cause and cardiovascular mortality (Tables S4–S6, Supplemental Digital Content, https://links.lww.com/MD/Q556).

### 3.4. Receiver operating characteristic (ROC) analysis: predictive value of RAR for all-cause and cardiovascular mortality in participants with CKM stages 1 to 4

**Figure 4.**
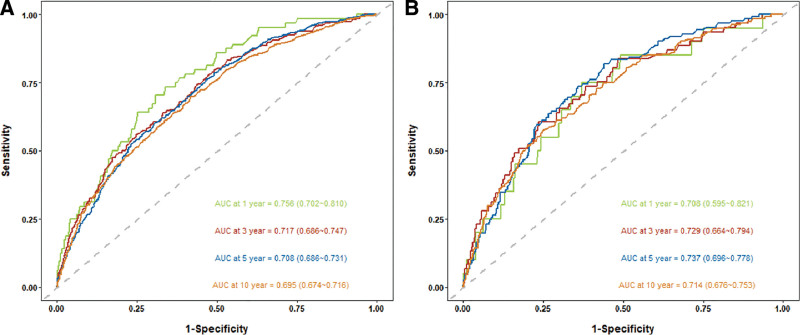
Time-dependent ROC curves of the RAR for predicting all-cause mortality (A) and cardiovascular mortality (B). RAR = red blood cell distribution width-to-albumin ratio, ROC = receiver operating characteristic.

Time-dependent ROC analysis was conducted to evaluate the prognostic value of RAR for all-cause and cardiovascular mortality in participants with CKM stages 1 to 4. The results revealed that the areas under the curves of the RAR for predicting all-cause mortality at 1, 3, 5, and 10 years were 0.756, 0.717, 0.708, and 0.695, respectively. The corresponding areas under the curves for cardiovascular mortality were 0.708, 0.729, 0.737, and 0.714 at 1, 3, 5, and 10 years, respectively (Fig. 4A and B). These findings suggest that the RAR has good predictive value for both short-term and long-term all-cause and cardiovascular mortality.

### 3.5. Competing risk analysis via the Fine–Gray model

**Figure 5.**
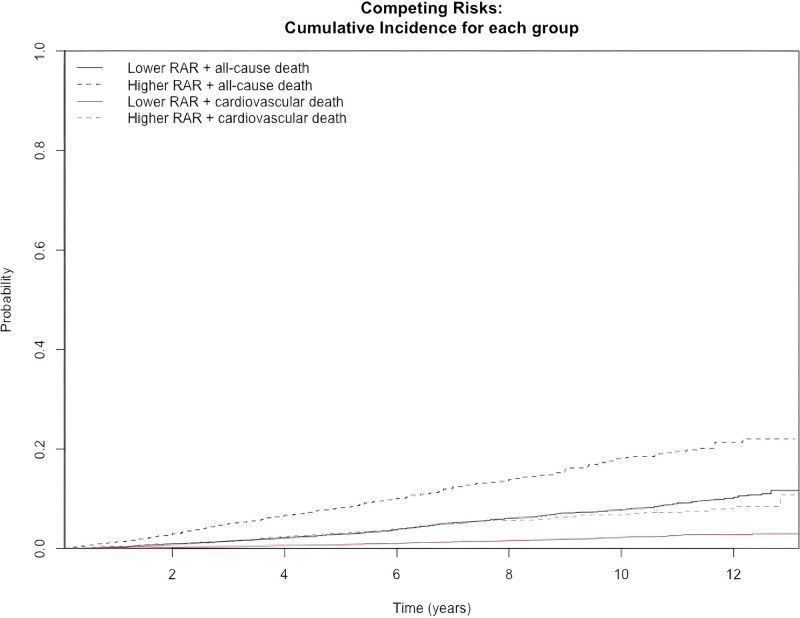
The association between RAR and cardiovascular death, with non-cardiovascular death as a competing risk (Fine–Gray competing risk model). RAR = red blood cell distribution width-to-albumin ratio.

To further explore the prognostic significance of RAR for cardiovascular mortality among participants with CKM stages 1 to 4, a competing risk analysis was performed via the Fine–Gray model, in which noncardiovascular deaths were treated as competing events. As shown in Figure 5, the Fine–Gray competing risk model indicated that the cumulative incidence of cardiovascular mortality was greater in the higher RAR group than in the lower RAR group. The subdistribution hazard function results from the Fine–Gray model are presented in Table 3. According to the fully adjusted model, RAR was significantly associated with cardiovascular mortality among CKM stage 1 to 4 participants (SHR 1.68, 95% CI 1.38–2.04, *P* < .0001). Compared with the lower RAR group, the higher RAR group presented a significantly increased risk of cardiovascular mortality (SHR 2.11, 95% CI 1.60–2.79, *P* < .0001).

**Table 3:** The associations between RAR and cardiovascular mortality among CKM stage 1 to 4 participants were further confirmed via the Fine–Gray competing risk model.

	Model 1 SHR (95% CI) *P*	Model 2 SHR (95% CI) *P*	Model 3 SHR (95% CI) *P*
RAR	1.92 (1.64–2.24) <.0001	2.02 (1.70–2.39) <.0001	1.68 (1.38–2.04) <.0001
RAR category			
Lower RAR	Reference	Reference	Reference
Higher RAR	3.33 (2.59–4.29) <.0001	2.80 (2.16–3.63) <.0001	2.11 (1.60–2.79) <.0001

Model 1: crude model without adjustment for any covariates. Model 2: adjustments for age, gender, and race; Model 3: adjustments for age, gender, race, marital status, education level, PIR, physical activity, smoking status, alcohol intake, BMI, waist circumference, hypertension, diabetes, CVD, stroke, and hyperlipidemia.

BMI = body mass index, CI = confidence interval, CKM = cardiovascular–kidney–metabolic, CVD = cardiovascular disease, RAR = red blood cell distribution width-to-albumin ratio, SHR = subdistribution hazard ratio.

## 4. Discussion

To the best of our knowledge, this is the first cohort study to evaluate the association between RAR and mortality in patients with CKM stages 1 to 4. When the population was stratified on the basis of the optimal RAR cutoff value of 3.33, which was determined by maximally selected rank statistics, Kaplan–Meier survival curves revealed that the cumulative incidence of mortality was significantly greater in the higher RAR group than in the lower RAR group. Subsequent weighted Cox regression analyses demonstrated a significant positive association between RAR and mortality across all the models, with individuals in the higher RAR group exhibiting markedly increased risks of both all-cause mortality and cardiovascular mortality. This positive association was further supported by GAMs, and the robustness and reliability of the findings were validated through multiple sensitivity analyses. Additionally, ROC curve analyses demonstrated that the RAR had good predictive performance for both all-cause mortality and cardiovascular mortality at 1, 3, 5, and 10 years. Finally, the results of the Fine–Gray competing risk model indicated that RAR remained significantly associated with cardiovascular mortality even after accounting for noncardiovascular deaths as competing events.

Obesity, diabetes, CKD, and cardiovascular disease constitute the core components of CKM syndrome. Substantial evidence has demonstrated that these conditions significantly increase the risk of both cardiovascular-specific mortality and all-cause mortality.^[28–31]^ In the present study, on the basis of clinical data from patients with CKM stages 1 to 4, we observed a significant positive association between RAR levels and mortality, which is consistent with findings from previous studies.^[22]^ The pivotal role of inflammation in disease progression and prognosis has gained increasing attention. In particular, among individuals with metabolic disorders (e.g., obesity and diabetes) and impaired renal function, chronic low-grade inflammation is considered a key contributor to the increased risk of cardiovascular events and mortality.^[32]^ Marialaura Bonaccio et al reported that chronic low-level inflammation is strongly associated with mortality among high-risk individuals, such as those with type 2 diabetes or preexisting cardiovascular disease.^[33]^ Sanchez et al reported that in diabetic patients, inflammatory status is an independent prognostic factor for cardiovascular death following an episode of unstable angina.^[34]^ Hoorn and colleagues further confirmed a positive association between low-grade systemic inflammation and increased cardiovascular mortality in the diabetic population.^[35]^ The relationship between inflammatory markers (e.g., IL-6) and mortality risk may be modulated by underlying disease conditions. Some studies have suggested that in individuals without a history of cardiovascular disease, this association becomes nonsignificant after adjusting for confounders.^[36]^

The potential mechanisms underlying the association between RAR and mortality are hypothesized to involve several key pathways. First, under systemic inflammatory conditions, sustained elevation of inflammatory cytokines may directly inhibit cardiac function.^[37,38]^ Second, inflammation plays a critical role in the development of atherosclerosis and plaque vulnerability. In patients with diabetes or cardiovascular disease, inflammation may accelerate lesion progression and trigger acute events.^[39]^ Third, inflammation-induced coagulation abnormalities may also serve as a crucial link between elevated RAR and increased mortality risk.^[33]^ The synergistic effects of these mechanisms may, at least in part, explain the observed positive association between RAR and mortality among patients with CKM stages 1 to 4.

Several limitations should be acknowledged. First, the observational design of this study limits the ability to infer causality. Although we identified an association between RAR and outcomes, a definitive causal relationship cannot be established. Second, this study was conducted among patients with CKM stages 1 to 4 in the United States; therefore, the generalizability of the findings to other populations remains to be determined. Third, the range of confounders adjusted for in our analysis may not have been comprehensive, and residual confounding that could influence the association between RAR and mortality cannot be excluded. Fourth, RAR was measured at a single time point, which may not capture changes over time or in response to interventions. Serial measurements of RAR may provide a more accurate representation of the inflammatory status. Fifth, since chronic inflammation is frequently associated with infection or thrombosis, and the NHANES cycles used in this study did not include data on monocyte distribution width or platelet distribution width, future prospective studies are encouraged to incorporate these parameters on top of RAR to allow for a more comprehensive assessment of the association between chronic inflammation and relevant outcomes. Finally, multiple comparisons correction (e.g., Bonferroni adjustment) was not performed in this study, which may increase the risk of type I error (false-positive findings).

In conclusion, our analysis of a large, nationally representative cohort demonstrated that elevated RAR is an independent predictor of increased risks of all-cause and cardiovascular mortality among individuals with CKM stages 1 to 4. These findings underscore the potential clinical utility of RAR as an inexpensive and widely accessible biomarker that could be incorporated into routine care to enhance risk stratification and prognostication in patients with CKM syndrome.

## Author contributions

**Conceptualization:** Bing Wang, Shanshan Zhou.

**Data curation:** Bing Wang, Shanshan Zhou.

**Formal analysis:** Bing Wang, Shanshan Zhou.

**Methodology:** Bing Wang, Shanshan Zhou.

**Project administration:** Shanshan Zhou.

**Resources:** Bing Wang, Shanshan Zhou.

**Software:** Bing Wang.

**Supervision:** Bing Wang, Shanshan Zhou.

**Validation:** Bing Wang, Shanshan Zhou.

**Visualization:** Bing Wang, Shanshan Zhou.

**Writing – original draft:** Bing Wang.

**Writing – review & editing:** Shanshan Zhou.

## Supplementary Material


