# Supplementary material for: Red blood cell distribution width-to-albumin ratio is a risk factor for all-cause and cardiovascular mortality in patients with CKM stages 1 to 4: Evidence from the NHANES 2007 to 2016
Source: Medicine (Baltimore). 2025 Nov 7;104(45):e45682. doi: 10.1097/MD.0000000000045682 (PMC12599703; doi:10.1097/MD.0000000000045682)
Supplement: Supplementary file 1 [file medi-104-e45682-s001.pdf]

Supplementary Materials Files

To: **Red blood Cell Distribution Width-to-Albumin Ratio is a Risk Factor for All-Cause and Cardiovascular Mortality in Patients with CKM Stages 1–4: Evidence from the NHANES 2007–2016**

by BingWang[1], Shanshan Zhou[1*]

Supplementary Method

Multiple imputation

Multiple imputation is a robust and widely used statistical technique for handling missing data across various fields, including epidemiology, social sciences, and clinical research. The method involves generating several plausible datasets through imputation, conducting separate analyses on each dataset, and pooling the results to produce valid estimates and measures of uncertainty[1,2]. This technique helps mitigate potential biases and ensures the representativeness of the findings.

The multiple imputation by chained equations (MICE) method, employed in this study, is a flexible approach that sequentially imputes missing values for each variable by developing regression models, conditioned on the observed values of other variables[1,2]. We created five imputed datasets and combined the results according to Rubin's Rules, ensuring the robustness and reliability of our conclusions.

Covariates

The demographic characteristics included age, race (non-Hispanic Black, non-Hispanic White, Mexican American, or other races), education level (less than high school, High school or equivalent, college or above)[3], the ratio of family income to the poverty line (low income (≤1.3), middle income (>1.3–3.5), and high income (>3.5))[4], and marital status (married, never married, living with partner,other)[3]. The health-related covariates included smoking status (Have you smoked at least 100 cigarettes in your entire life?)[5], alcohol consumption (frequency of drinking more than 12 times in the past year)[4], physical activity (participants were considered physically active if they engaged in moderate/vigorous work/recreational activities)[6], body mass index (BMI, weight divided by height squared)[7], waist circumference, and eGFR was calculated using the 2021 race and ethnicity- free Chronic Kidney Disease Epidemiology Collaboration creatinine equation[8]. Hypertension was defined as currently taking prescription medication for hypertension, having been told by a doctor at least twice that they had hypertension, or having an average systolic blood pressure of ≥140 mmHg and an average diastolic blood pressure of ≥90 mmHg on three consecutive measurements[9]. Diabetes (any of the following: diagnosed by a doctor, HbA1c (%) ≥6.5, fasting blood glucose (mg/dl) ≥126, use of diabetes medications or insulin)[10], and cardiovascular diseases (CVD) was defined as a self-reported "yes" response to any of the following conditions: coronary heart disease, angina, angina pectoris, or heart attack. Stroke was defined as a physician-diagnosed condition based on a "yes" response to the relevant questionnaire item[11]. Hyperlipidemia was defined as having total cholesterol levels of ≥200 mg/dL, triglyceride levels of ≥150 mg/dL, low-density lipoprotein levels of ≥130 mg/dL, or high-density lipoprotein levels of ≤50 mg/dL for women and ≤40 mg/dL for men. Alternatively, individuals who acknowledged the use of cholesterol-lowering medication were also classified as having hyperlipidemia[12].

References:

1. Beesley LJ, Bondarenko I, Elliot MR, Kurian AW, Katz SJ, Taylor JM. Multiple imputation with missing data indicators. *Stat Methods Med Res*. Dec 2021;30(12):2685-2700. doi:10.1177/09622802211047346

2. White IR, Royston P, Wood AM. Multiple imputation using chained equations: Issues and guidance for practice. *Stat Med*. Feb 20 2011;30(4):377-99. doi:10.1002/sim.4067

3. Liu J, Huang S. Dietary index for gut microbiota is associated with stroke among US adults. *Food Funct*. Feb 17 2025;16(4):1458-1468. doi:10.1039/d4fo04649h

4. Ruan Z, Lu T, Chen Y, et al. Association Between Psoriasis and Nonalcoholic Fatty Liver Disease Among Outpatient US

Adults. *JAMA Dermatol*. Jul 1 2022;158(7):745-753. doi:10.1001/jamadermatol.2022.1609
5. Zhang X, Wei R, Wang X, et al. The neutrophil-to-lymphocyte ratio is associated with all-cause and cardiovascular mortality among individuals with hypertension. *Cardiovasc Diabetol*. Apr 2 2024;23(1):117. doi:10.1186/s12933-024-02191-5
6. Tan L, Zhou Q, Liu J, Liu Z, Shi R. Association of iron status with non-alcoholic fatty liver disease and liver fibrosis in US adults: a cross-sectional study from NHANES 2017-2018. *Food Funct*. Jun 19 2023;14(12):5653-5662. doi:10.1039/d2fo04082d
7. Wang L, Yi J, Guo X, Ren X. Associations between life's essential 8 and non-alcoholic fatty liver disease among US adults. *J Transl Med*. Dec 23 2022;20(1):616. doi:10.1186/s12967-022-03839-0
8. Inker LA, Eneanya ND, Coresh J, et al. New Creatinine- and Cystatin C-Based Equations to Estimate GFR without Race. *The New England journal of medicine*. Nov 4 2021;385(19):1737-1749. doi:10.1056/NEJMoa2102953
9. Drew DA, Katz R, Kritchevsky S, et al. Soluble Klotho and Incident Hypertension. *Clin J Am Soc Nephrol*. Oct 2021;16(10):1502-1511. doi:10.2215/cjn.05020421
10. Tan L, Liu Y, Liu J, Zhang G, Liu Z, Shi R. Association between insulin resistance and uncontrolled hypertension and arterial stiffness among US adults: a population-based study. *Cardiovasc Diabetol*. Nov 9 2023;22(1):311. doi:10.1186/s12933-023-02038-5
11. Jiang Y, Shen J, Chen P, et al. Association of triglyceride glucose index with stroke: from two large cohort studies and Mendelian randomization analysis. *Int J Surg*. Sep 1 2024;110(9):5409-5416. doi:10.1097/js9.0000000000001795
12. Yan S, Luo W, Lei L, Zhang Q, Xiu J. Association between serum Klotho concentration and hyperlipidemia in adults: a cross-sectional study from NHANES 2007-2016. *Front Endocrinol (Lausanne)*. 2023;14:1280873. doi:10.3389/fendo.2023.1280873
13. KDIGO 2024 Clinical Practice Guideline for the Evaluation and Management of Chronic Kidney Disease. *Kidney international*. Apr 2024;105(4s):S117-s314. doi:10.1016/j.kint.2023.10.018
14. Whelton PK, Carey RM. The 2017 American College of Cardiology/American Heart Association Clinical Practice Guideline for High Blood Pressure in Adults. *JAMA cardiology*. Apr 1 2018;3(4):352-353. doi:10.1001/jamacardio.2018.0005
15. 2. Classification and Diagnosis of Diabetes: Standards of Medical Care in Diabetes-2022. *Diabetes care*. Jan 1 2022;45(Suppl 1):S17-s38. doi:10.2337/dc22-S002
16. Khan SS, Matsushita K, Sang Y, et al. Development and Validation of the American Heart Association's PREVENT Equations. *Circulation*. Feb 6 2024;149(6):430-449. doi:10.1161/circulationaha.123.067626

Table S1. The basic PREVENT 10-year risk estimation model equations

10-year CVD risk assessment equation	
**Men**	**log-Odds** = -3.031168 + 0.7688528 × (age – 55) /10 + 0.0736174 × ((TC – HDL-C) × 0.02586 – 3.5) – 0.0954431 × (HDL-C × 0.02586 – 1.3) /0.3 – 0.4347345 × (min(SBP, 110) – 110) /20 + 0.3362658 × (max(SBP, 110) – 130) /20 + 0.7692857 × (if diabetes) + 0.4386871 × (if current smoker) + 0.5378979 × (min(eGFR, 60) – 60) / -15 + 0.0164827 × (max(eGFR, 60) – 90) / -15 + 0.288879 × (if using anti hypertensive medication) – 0.1337349 × (if using statin) – 0.0475924 × (if using anti-hypertensive medication) × (max(SBP, 110) – 130) /20 + 0.150273 × (if using statin) × ((TC – HDL-C) × 0.02586 – 3.5) – 0.0517874 × (age – 55) /10 × ((TC – HDL-C) × 0.02586 – 3.5) + 0.0191169 × (age – 55) /10 × (HDL-C × 0.02586 – 1.3) /0.3 – 0.1049477 × (age – 55) /10 × (max(SBP, 110) – 130) /20 – 0.2251948 × (age – 55) /10 × (if diabetes) – 0.0895067 × (age – 55) /10 × (if current smoker) – 0.1543702 × (age – 55) /10 × (min(eGFR, 60) – 60) / -15
	**Risk**= 1 / (1 + exp(-log-Odds))
**Women**	log-Odds = -3.307728 + 0.7939329 × (age – 55) /10 + 0.0305239 × ((TC – HDL-C) × 0.02586 – 3.5) – 0.1606857 × (HDL-C × 0.02586 – 1.3) /0.3 – 0.2394003 × (min(SBP, 110) – 110) /20 + 0.360078 × (max(SBP, 110) – 130) /20 + 0.8667604 × (if diabetes) + 0.5360739 × (if current smoker) + 0.6045917 × (min(eGFR, 60) – 60) / -15 + 0.0433769 × (max(eGFR, 60) – 90) / -15 + 0.3151672 × (if using anti hypertensive medication) – 0.1477655 × (if using statin) – 0.0663612 × (if using anti-hypertensive medication) × (max(SBP, 110) – 130) /20 + 0.1197879 × (if using statin) × ((TC – HDL-C) × 0.02586 – 3.5) – 0.0819715 × (age – 55) /10 × ((TC – HDL-C) × 0.02586 – 3.5) + 0.0306769 × (age – 55) /10 × (HDL-C × 0.02586 × 0.02586 – 1.3) /0.3 – 0.0946348 × (age – 55) /10 × (max(SBP, 110) – 130) /20 – 0.27057 × (age – 55) /10 × (if diabetes) – 0.078715 × (age – 55) /10 × (if current smoker) – 0.1637806 × (age – 55) /10 × (min(eGFR, 60) – 60) / -15
	**Risk**= 1 / (1 + exp(-log-Odds))

Abbreviations: TC, total cholesterol; HDL-C, high-density lipoprotein cholesterol; SBP, systolic blood pressure; eGFR, estimated glomerular filtration rate.

Table S2. Definition of CKM syndrome staging adjusted for data in NHANES

CKM syndrome stages	Definition
Stage 0: No CKM health risk factors	Individuals without overweight/obesity, metabolic risk factors (hypertriglyceridemia, hypertension, diabetes, MeTS), CKD or subclinical/clinical CVD<br>(1)BMI between 18.5 and 25 kg/m^2, inclusive<br>(2)WC <102 cm for men or <88 cm for women
Stage 1: Excess or dysfunctional adiposity	Individuals with overweight/obesity, abdominal obesity, or adipose tissue dysfunction without other metabolic risk factors, CKD, or subclinical/clinical CVD<br>(1)BMI ≥ 25 kg/m^2<br>(2)WC ≥ 102 cm for men or ≥ 88 cm for women<br>(3)FBG levels ranging from 100 to 124 mg/dL, or HbA1c levels between 5.7 and 6.4 %
Stage 2: Metabolic risk factors and moderate to high-risk CKD	Individuals with metabolic risk factors (hypertriglyceridemia, hypertension, diabetes, MeTS*) or moderate to high-risk CKD stage(The stage of CKD is determined by the KDIGO criteria, using eGFR and UACR[13]. The eGFR was calculated using the 2021 race and ethnicity□free Chronic Kidney Disease Epidemiology Collaboration creatinine equation[8].)<br>(1)TG >135 mg/dL<br>(2)Hypertension is defined by an SBP of ≥130 mm Hg, a DBP of ≥80 mm Hg, a medical diagnosis, or taking antihypertensive medication[14].<br>(3)Diabetes is defined by FBG levels of > 126 mg/dL, HbA1c levels of ≥ 6.5%, a medical diagnosis, or taking insulin or glucose-lowering medication[15].<br>(4) Moderate to high-risk CKD in the KDIGO classification is defined as UACR ≥ 30 mg/g and eGFR ≥ 60 $ml/min/1.73m^2$, UACR < 300 mg/g and eGFR ≤ 45-59 $ml/min/1.73m^2$, or UACR < 30 mg/g and eGFR ≤ 30-44 $ml/min/1.73m^2$.
Stage 3: Subclinical CVD in CKM	Risk equivalents for subclinical CVD: high predicted 10-year CVD risk or very high-risk KDIGO CKD stage<br>(1)A high 10-year CVD risk is defined as a 20% or above risk, as determined by the basic Predicting Risk of CVD EVENTs (PREVENT) equation[16].<br>(2)Very high-risk CKD in the KDIGO classification is defined as UACR ≥ 300 mg/g and eGFR ≤ 45-59 $ml/min/1.73\ m^2$, UACR ≥ 30 mg/g and eGFR ≤ 30-44 $ml/min/1.73\ m^2$, or eGFR ≤ 29 $ml/min/1.73\ m^2$.
Stage 4: Clinical CVD in CKM	Clinical CVD (self-reported diagnosed cardiovascular disease, including heart failure, coronary heart disease, angina, heart attack, and stroke) in individuals

Abbreviations: CKM syndrome, Cardiovascular-Kidney-Metabolic syndrome; NHANES, National Health and Nutrition Examination Survey; MeTS, metabolic syndrome; CKD, chronic kidney disease; CVD, cardiovascular disease; BMI, body mass index; WC, waist circumference; FBG, fasting blood glucose; HbA1c, glycated hemoglobin A1c; KDIGO, Kidney Disease Improving Global Outcomes; UACR, urine albumin-to-creatinine ratio; eGFR, estimated glomerular filtration rate; TG, triglycerides; SBP, systolic blood pressure; DBP, diastolic blood pressure.

* MeTS is defined by the presence of ≥3 of the following: (1) WC ≥ 102 cm for men or ≥ 88 cm for women; (2) HDL-C <40 mg/dL for men, <50 mg/dL for women; (3) TG ≥150 mg/dL; (4) Elevated blood pressure (SBP ≥130 mm Hg, DBP ≥80 mm Hg, a medical diagnosis, or taking antihypertensive medication); (5) FBG ≥100 mg/dL

Table S3. Subgroup analysis

Characteristics	Lower RAR (≤ 3.43)	Higher RAR[#] (> 3.43) HR (95% CI)	$p^{\#}$	p for interaction	Higher RAR[*] (> 3.43) HR (95% CI)	p^{*}	p for interaction
Age				0.3742			0.6589
≤60	Reference	2.22 (1.62, 3.03)	<0.0001		2.29 (1.14, 4.61)	0.0200	
>60	Reference	2.00 (1.71, 2.33)	<0.0001		2.74 (2.02, 3.71)	<0.0001	
Gender				0.3742			0.6589
Female	Reference	1.93 (1.56, 2.37)	<0.0001		2.42 (1.54, 3.81)	0.0001	
Male	Reference	2.20 (1.83, 2.65)	<0.0001		3.09 (2.15, 4.42)	<0.0001	
Smoking status				0.9289			0.1897
No	Reference	2.09 (1.67, 2.61)	<0.0001		2.35 (1.55, 3.58)	<0.0001	
Yes	Reference	2.12 (1.78, 2.52)	<0.0001		3.29 (2.25, 4.81)	<0.0001	
BMI				0.5420			0.4488
≤25	Reference	2.18 (1.70, 2.79)	<0.0001		3.12 (1.79, 5.44)	0.0028	
25-30	Reference	2.21 (1.74, 2.81)	<0.0001		3.21 (2.00, 5.16)	<0.0001	
>30	Reference	2.06 (1.49, 2.39)	<0.0001		2.18 (1.39, 3.42)	<0.0001	
Hypertension				0.9163			0.2568

No	Reference	2.36 (1.80, 3.09)	<0.0001		2.47 (1.36, 4.47)	0.0028	
Yes	Reference	2.00 (1.70, 2.34)	<0.0001		2.67 (1.95, 3.67)	<0.0001	
Diabetes				0.4534			0.7775
No	Reference	2.09 (1.75, 2.49)	<0.0001		3.01 (2.09, 4.33)	<0.0001	
Yes	Reference	2.06 (1.80, 2.37)	<0.0001		2.38 (1.54, 3.68)	<0.0001	

RAR: ratio of red cell distribution width to albumin; HR: hazard ratio; CI: confidence interval

[#]All-cause mortality; *cardiovascular mortality. adjustments for age, gender, race, marital status, pir, education levels, physical activity, smoking status, alcohol intake,bmi, waist circumference, egfr, hypertension, diabetes, cvd, stroke and hyperlipidemia.

Table S4. Association between RAR and mortality of the NHANES 2007-2016 participants after multiple imputation

	Model1<br>HR (95% CI) *P*	Model2<br>HR (95% CI) *P*	Model3<br>HR (95% CI) *P*
All-cause mortality			
RAR	2.22 (2.00, 2.46) <0.0001	2.88 (2.47, 3.35) <0.0001	2.04 (1.84,2.26) <0.0001
RAR category			
Lower RAR	Reference	Reference	Reference
Higher RAR	3.33 (2.86,3.87) <0.0001	2.72 (2,34,3,17) <0.0001	2.10 (1.83,2.41) <0.0001
Cardiovascular mortality			
RAR	2.36 (2.04, 2.72) <0.0001	2.65 (2.26, 3.11) <0.0001	2.17 (1.81, 2.61) <0.0001
RAR category			
Lower RAR	Reference	Reference	Reference
Higher RAR	4.46 (3.61,5.51) <0.0001	3.76 (2.93,4.81) <0.0001	2.57 (1.99,3.34) <0.0001

RAR: ratio of red cell distribution width to albumin; HR: hazard ratio; CI: confidence interval

Model 1: unadjusted for any covariates.

Model 2: adjusted for age and race.

Model 3: adjusted for age, gender, race, marital status, pir, education levels, physical activity, smoking status, alcohol intake,bmi, waist circumference, egfr, hypertension, diabetes, cvd, stroke and hyperlipidemia.

Table S5. Unweighted association between RAR and mortality of the NHANES 2007-2016 participants

	Model1<br>HR (95% CI) *P*	Model2<br>HR (95% CI) *P*	Model3<br>HR (95% CI) *P*
All-cause mortality			
RAR	1.20 (1.18, 1.22) <0.0001	1.17 (1.14, 1.19) <0.0001	1.88 (1.72,2.05) <0.0001
RAR category			
Lower RAR	Reference	Reference	Reference
Higher RAR	1.99 (1.87,2.13) <0.0001	2.57 (2.26,2.94) <0.0001	2.07 (1.80,2.37) <0.0001
Cardiovascular mortality			
RAR	2.09 (1.85, 2.35) <0.0001	2.29 (1.99, 2.63) <0.0001	2.02 (1.71,2.39) <0.0001
RAR category			
Lower RAR	Reference	Reference	Reference
Higher RAR	3.59 (2.79,4.63) <0.0001	3.37 (2.59,4.40) <0.0001	2.70 (2.04,3.57) <0.0001

RAR: ratio of red cell distribution width to albumin; HR: hazard ratio; CI: confidence interval

Model 1: unadjusted for any covariates.

Model 2: adjusted for age and race.

Model 3: adjusted for age, gender, race, marital status, pir, education levels, physical activity, smoking status, alcohol intake,bmi, waist circumference, egfr, hypertension, diabetes, cvd, stroke and hyperlipidemia.

Table S6. Association Between RAR Quartiles and All-Cause Mortality in US Adults: A Population-Based Study from NHANES 2007-2016

	Model1<br>HR (95% CI) *P*	Model2<br>HR (95% CI) *P*	Model3<br>HR (95% CI) *P*
All-cause mortality			
RAR category			
Q1	Reference	Reference	Reference
Q2	1.77 (1.31,2.39) 0.0002	1.34 (0.98,1.83) 0.0670	1.30 (0.96,1.78) 0.0933
Q3	2.59 (2.04,3.29) <0.0001	1.74 (1.34,2.26) <0.0001	1.49 (1.15,1.93) 0.0027
Q4	5.67 (4.45,7.22) <0.0001	4.02 (3.08,5.23) <0.0001	3.01 (2.33,3.87) <0.0001
Cardiovascular mortality			
RAR category			
Q1	Reference	Reference	Reference
Q2	1.87 (1.02,3.44) 0.0423	1.39 (0.76,2.53) 0.2811	1.34 (0.75,2.42) 0.3250
Q3	3.15 (1.94,5.11) <0.0001	2.06 (1.25,3.38) 0.0044	1.66 (1.00,2.76) 0.0489
Q4	8.61 (5.22,14.20) <0.0001	6.20 (3.52,10.91) <0.0001	4.49 (2.61,7.72) <0.0001

Model 1: unadjusted for any covariates.

Model 2: adjusted for age and race.

Model 3: adjusted for age, gender, race, marital status, pir, education levels, physical activity, smoking status, alcohol intake,bmi, waist circumference, egfr, hypertension, diabetes, cvd, stroke and hyperlipidemi

Figure S1 Flow Chart

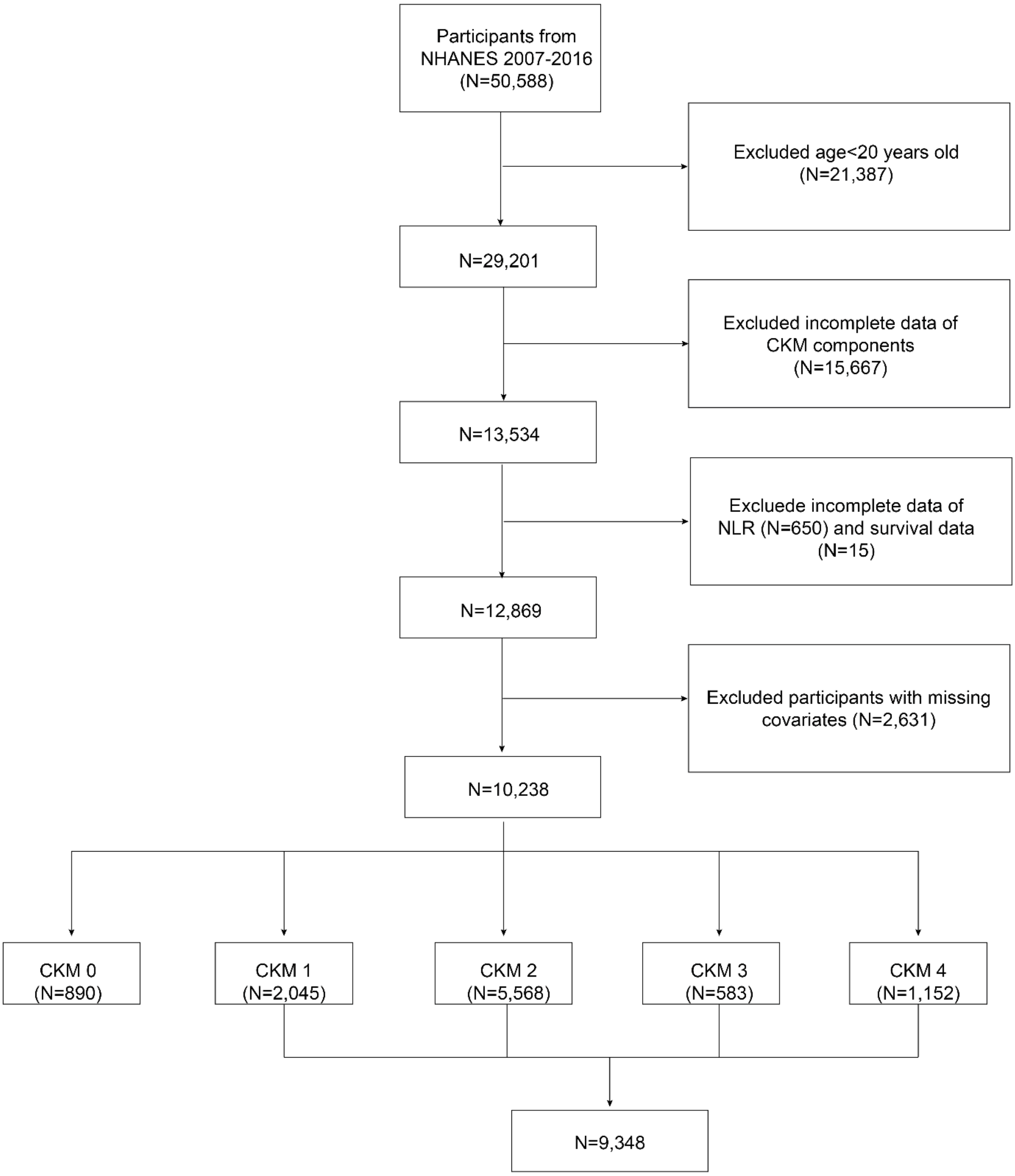

Abbreviations: NHANES: National Health and Nutrition Examination Survey. CKM: Cardiovascular–kidney–metabolic syndrome.
